# Cultural isolation of spore-forming bacteria in human feces using bile acids

**DOI:** 10.1038/s41598-020-71883-1

**Published:** 2020-09-14

**Authors:** Masaru Tanaka, Sakura Onizuka, Riko Mishima, Jiro Nakayama

**Affiliations:** Laboratory of Microbial Technology, Division of Systems Bioengineering, Department of Bioscience and Biotechnology, Faculty of Agriculture, Graduate School, Kyushu University, Fukuoka, Japan

**Keywords:** Bacterial techniques and applications, Microbial communities, Microbiome

## Abstract

Structurally-diversified bile acids (BAs) are involved in shaping of intestinal microbiota as well as absorption of dietary lipids. Taurocholic acid, a conjugated form of BA, has been reported to be a factor triggering germination of a wide range of spore-forming bacteria in intestine. To test a hypothesis that other BAs also promote germination of intestinal bacteria, we attempted culture of bacteria from ethanol-treated feces by using a series of BAs. It was found that conjugated-BAs, notably three glycine-conjugated BAs, glycodeoxycholic acid and glycochenodeoxycholic acid, significantly increased the number and the species variety of colonies formed on the agar plate. These colonized bacteria mostly belonged to class Clostridia, mainly consisting of families Lachnospiraceae, Clostridiaceae, and Peptostreptococcaceae. There were several types of bacteria associated with different sensitivity to each BA. Eventually, we isolated 72 bacterial species of which 61 are known and 11 novel. These results demonstrate that the culturable range of bacteria in intestine can be widened using the germination-inducing activity of BAs. This approach would advance the research on spore-forming Clostridia that contains important but difficult-to-cultured bacteria associate with host health and diseases.

## Introduction

The crosstalks of host-gut microbes interaction plays a crucial role in the health and disease of human being^1^. The number of bacterial cells residing in the body of human is estimated to be approximately 40-trillion bacterial cells^2^, consisting of several hundreds of bacterial species out of total 4,500 estimated for all human on the earth^3^. More than 2,600 of 4,500 species are estimated to be uncultured and have never been isolated in the laboratory^3,4^, despite including members that are believed to play an important biological role that remains undiscovered^2,5^. Although several metagenomic studies shed light on these uncultured bacteria that may have avoided past culture strategies^6^, a new wave of gut microbiota culturomics is decreasing the number of unclassified microbial diversity within the gut ecosystem^7–12^.

The phylum Firmicutes, which includes Lachnospiraceae, Ruminococcaceae and Clostridiaceae*,* accounts for approximately half of the gut microbial community, many of which are known as spore-forming bacteria^7,8,13,14^. The high tolerance of these spore-forming bacteria to gut environment stress such as high and low temperature, nutrient and oxygen and drug attack allows their long-term survival^7,14–16^. It is also known that spores tolerant to the outside gut environment facilitate the host-to-host transmission of bacteria through spatio-temporal means, which is not limited to pathogens, but includes commensals beneficial to health^14^.

Bile stress is one of the stresses experienced by intestinal bacteria. In humans, there are two main BAs, cholic acid (CA) and chenodeoxycholic acid (CDCA), which are produced and secreted into the small intestine as taurine- or glycine-conjugated forms to aid lipid absorption by forming water-soluble micelles^17,18^. BAs are mostly reabsorbed and recycled at the end of the small intestine, and thereafter parts of them passed through to the large intestine, after which they are processed to secondary BAs by large intestinal microbiota. Specifically, conjugated BAs are deconjugated by the function of the bacterial bile salt hydrolase (BSH)^19,20^ and then converted to secondary BAs through 7α-dehydroxylation, resulting in deoxycholic acids (DCA) and lithocholic acid (LCA) from CA and CDCA respectively. Alternatively, 7α-epimerization results in ursodeoxycholic acid (UDCA) from CDCA^21–23^. Since these BA converting enzymes are unique to certain species of bacteria, the final composition of BAs will differ depending on the composition of gastro-intestinal microbiota^24^.

With bactericidal activity, BAs provide selective pressure for the colonization of bile-sensitive bacteria^25^. In contrast, some spore-forming bacteria including pathogens and commensals, germinate in response to BAs in the intestinal tract^7^. The germination of *Clostridioides difficile* spores, the pathogen that causes diarrhea, is stimulated by a combination of nutrients and bile salts present in the gastrointestinal tract, specifically taurocholic acid (TCA)^26–29^. It was recently reported that TCA also induces germination of certain populations of spore-forming bacteria in the intestine and expands the culturability of gut bacteria^7^.

Here, we hypothesize that many spore-forming bacteria in intestinal tract control germination by using TCA as the environmental signal. To address this hypothesize, we demonstrated the germination-inducing activity of a series of human BAs against spore-formers in feces, which are expected to include a number of yet-to-be cultured bacterial species.

## Results

### Germination of spores in feces by using various BAs

**Figure 1:**
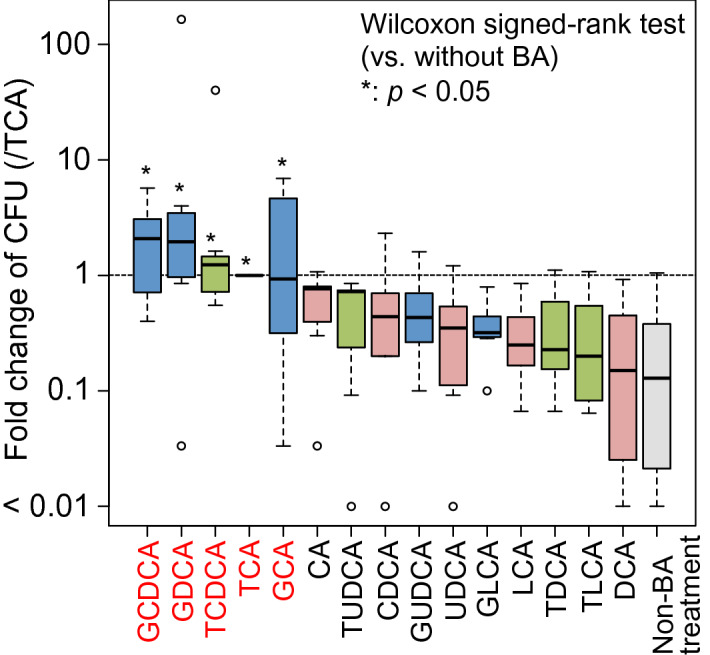
Fold change of cfu of ethanol-treated fecal sample by a series of BAs. The number of colonies formed on agar plates containing each BA is expressed as a fold change with respect to the BA positive control (TCA). A paired Wilcoxon signed-rank test was used to test the significance of the difference in the fold change to the negative control non-BA treatment (*p < 0.05).

To examine the activity of a series of BAs on spore germination, seven fresh fecal samples were treated with an equal volume of 70% (v/v) ethanol to kill vegetative cells. Surviving spores were treated with each BA then spread and culture on GAM agar medium. For a negative control, non-BA treated spores were also spread and culture on the plate in the same manner. The number of colonies formed from samples treated by each BA signatures was counted individually for the seven fecal samples and statistically compared with the different BA groups (Table S1). The colony forming units (cfu) of the ethanol untreated samples was about 10,000 times that of the ethanol treated sample. As shown in Fig. 1, the cfu. were significantly higher in the samples treated by glycodeoxycholic acid (GDCA), glycochenodeoxycholic acid (GCDCA), taurochenodeoxycholic acid (TCDCA), TCA and glycocholic acid (GCA) compared to the BA-untreated sample, suggesting that these BAs have germination inducing activity for spore-forming bacteria present in the intestine (*p* < 0.05, Wilcoxon signed-rank test). GCDCA and GDCA in particular, showed approximately two-fold higher activity compared to TCA, which is a known germination inducer, although not statistically significant (*p* > 0.375, Wilcoxon signed-rank test).

### Spectrum of germination-inducing activity of the human BAs

**Figure 2:**
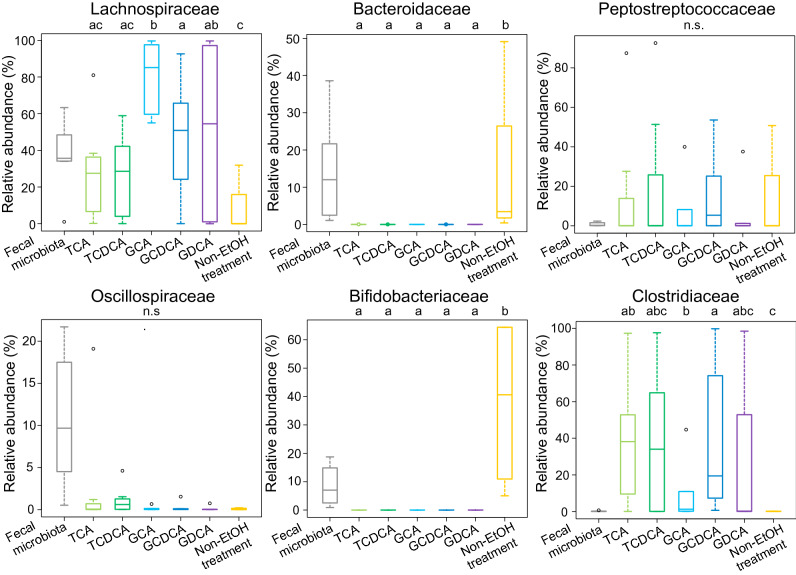
The effect of the series of BAs on the colonization of each family. The fecal samples from 7 adults were treated by the series of BAs following ethanol treatment and were subjected to the plate culture. For the control to capture culturable vegetative cells of non-spore-forming and spore-forming bacteria, fecal sample was directly cultured on the medium without ethanol and BA treatments. Colonies on the plate were pooled and subjected to the 16S rRNA gene amplicon sequencing. Also, to capture the microbiome of original fecal samples, total bacterial DNA was extracted from fecal samples and subjected to the 16S rRNA gene amplicon sequencing. OTUs were generated from the obtained 16S rRNA gene V3-V4 region sequences. Box plots show the distribution of the relative abundance of bacterial family among the data from seven donors. Different letters (a-c) indicate significant differences between treated BA signatures (p < 0.05, pair-wise Mann Whitney *U* test). *n.s.* non-significantly.

To assess the population and the phylogenetic variety of intestinal bacteria responsive to the 15 BAs, the bacterial cells of colonies formed on each BA plate were collected and subjected to 16S rRNA gene amplicon sequencing. For the control, colonies from fecal samples without ethanol treatments, which represented cultivable vegetative cells of non-spore-forming and spore-forming bacteria, were analyzed in the same manner. The relative abundance of each bacterial family was determined for each plate. The data obtained from fecal samples of 7 donors were statistically compared among each BA-treated groups and the non-ethanol treated control group (Fig. 2). Neither Bacteroidaceae and Bifidobacteriaceae were observed in the ethanol-BA treated samples while their colonization was confirmed on the plate of non-ethanol treated control, confirming that the ethanol treatment killed these non-spore-forming bacteria of these families. On the other hand, families of Clostridiaceae, Lachnospiraceae and Peptostreptococcaceae were enriched in the ethanol-BA treated samples. Particularly, the relative abundance of Lachnospiraceae was significantly higher in the samples treated by GCA compared to those treated by other BAs.

**Figure 3:**
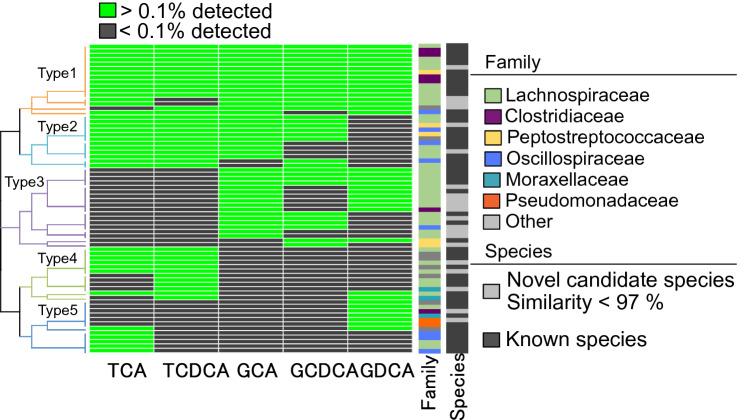
Heat map of detected OTUs in each BA-treated sample. OTUs whose abundances were higher than 0.1% on each plate were selected and then clustered according to their abundance in each BA-treated sample. Each cell shows by color, the relative abundance to total population (black, < 0.1%; green, 0.1% >). The taxonomic classification at family level was displayed in the “Family” column with colors representing the bacterial taxonomies at the bottom panel. The OTUs were also annotated at species level, while novel candidate species were listed with 16S rRNA gene sequences showing less than 97% identity with any described species (black in the “Species” column).

The heat map in Fig. 3 shows the distribution of OTUs observed from each BA-treated sample and their annotation to bacterial families and species. Colored cells indicate the presence of OTUs among the colonies cultured from each BA-treated sample. These OTUs were classified into 12 families, constituting mainly of Lachnospiraceae, Clostridiaceae, Peptostreptococcaceae, and Oscillospiraceae. Further, these OTUs were clustered into five types based on the responsiveness to the different BAs. Type-1 OTUs were detected from all BA-treated samples. Type-2 OTUs were commonly detected from the samples treated by conjugated primary BAs. Type 3 and Type 4 OTUs were detected from the samples treated by glycine-conjugated BAs or taurine-conjugated BAs respectively. Type 5 OTUs were detected from the samples specifically treated by TCA or GDCA. A number of OTUs, particularly from the samples treated by glycine-conjugated BAs, were not annotated with any known species (similarity < 97%), suggesting the effective enrichment of as-yet-uncultured species whose germination is triggered by glycine conjugated BA^30^.

### Taxonomy of cultured isolates

We cultured 438 bacterial colonies isolated from the ethanol-BA treated fecal samples of seven donors, as well as 67 colonies from untreated feces of the same donors (Table S2). Theses isolates were subjected to taxonomy analysis using full-length 16S rRNA gene sequences. The derived 16S rRNA gene sequences were dereplicated into 94 OTUs with more than 98.7% sequence identity. The number of the MiSeq sequence reads mapped to these 94 OTUs accounted for 49.9% of total amplicon reads from fecal microbiotas of the seven subjects, suggesting that approximately half portion of human gastro-intestinal microbiota was successfully cultured.

**Figure 4:**
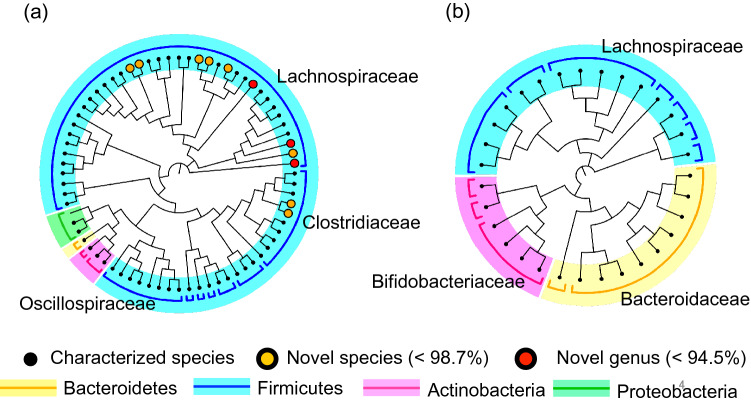
Phylogenetic tree of bacterial isolates from seven donors. Two trees were constructed by the neighbor joining method using the full-length 16S rRNA gene sequences of 72 OTUs of colony isolates from ethanol-BA treated feces of the seven donors (**a**) and 30 OTUs from untreated feces from the same donors (**b**). Novel candidate genera and species are marked with red and orange dots, respectively. Major phyla and family names are indicated. More details of OTUs are tabulated in Table S1.

Each OTU was taxonomically annotated by comparison against the EzBioCloud 16S rRNA gene database. Consequently, 94 OTUs were classified into four main phyla of human intestinal bacteria, namely Firmicutes, Bacteroidetes, Actinobacteria, and Proteobacteria (Fig. 4). Seventy-two OTUs observed from the ethanol-BA treated fecal samples belonged primarily to families Lachnospiraceae, Clostridiaceae and Oscillospiraceae, whereas 30 OTUs from the untreated samples mainly belonged to the Bacteroidaceae and Bifidobacteriaceae families. Out of 94 OTUs, 7 were observed from both treated and untreated. The taxonomy of each OTU was identified at the species level with more than 98.7% identity and at genus level with more than 94.5% identity to the 16S rRNA gene sequence of the closest species in the database^31^. Consequently, 81 known species were identified, while 11 OTUs, all of which were uniquely isolated from the ethanol-BA treated samples, were not identified to any known species in the database. Further, of the 11 OTUs, 3 were recognized as novel candidate genus. These candidates belong to either the Lachnospiraceae or Clostridiaceae families of the Firmicutes phylum.

### Specificity in the responsiveness of spore-forming bacteria to BAs

**Figure 5:**
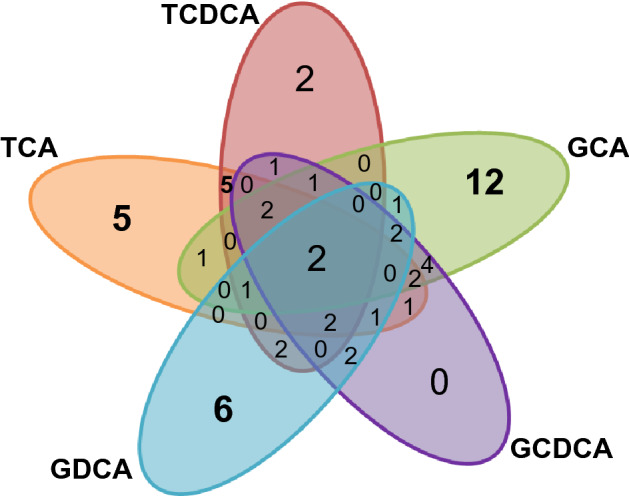
Venn diagrams showing the number of shared and unshared OTUs among the different BA-treated samples. The 72 OTUs of the colony isolates were subjected to count the number of shared and unique OTUs.

Figure 5 shows the number of shared and unshared OTUs among the different BA-treated samples. Glycine-conjugated BAs offered somewhat higher number of unique OTUs compared to taurine-conjugated BAs. Notably, 4 and 3 OTUs belonging to genera *Roseburia* and *Blautia*, respectively, were obtained only from the samples treated by glycine-conjugated BAs (Table S2). Furthermore, from glycine-conjugated BAs, 10 novel candidate species were obtained, while only 2 was found from taurine-conjugated BAs. These results suggest the specificity in the responsiveness of spore-forming bacteria to BAs as well as validity of glycine-conjugated BAs to culture uncultured species of human gastro-intestinal tract.

## Discussion

In this study, we focused on the germination-inducing activity of a series of human BAs against spore-forming bacteria in human gut microbiota and found that spores in human feces are largely responsive to BAs. The induction of germination depends on the BA signatures, conjugated BAs in particular. Two types of conjugated BAs were tested in this study, namely taurine- and glycine-conjugated BAs. These showed rather strong activity, in accordance with a previous study which showed the efficacy of TCA^6^ notably for uncultured intestinal bacteria, as well as *C. difficile* whose spore formation is problematic to the antibiotic-associated colitis patients^26–29^. The present study showed that glycine conjugated BAs showed a novel germination-inducing capability in terms of effective range of target species including 9 yet-to-be cultured species belonging to class Clostridia.

Our results showed that human intestinal clostridia tend to be more sensitive to glycine-conjugated BAs than TCA. The difference in the sensitivity spectra may be related to different intestinal microbial niche, such as taurine-conjugate^32^ rich mouse intestine, glycine-conjugate rich adult human intestine^33^, and human infant’s intestine lacking in glycine-conjugates^24^.

It is known that tere are complex machineries equipped in spores, allowing their germination in response to multiple environmental signals including not only bile acids but also nutrient signals such as amino acids, nucleic acids, and sugars. The systems are studied well in clostridial pathogens such as *C. difficile*, *C. perfringens*, and *C. botulinum*, in which they share common cascade signaling system with three Csp proteins^34^. These Csp proteases are also conserved in many Clostridia organisms, such as Clostridiaceae, Lachnospiraceae and Peptostreptococcaceae family^35^. However, germinant receptors are diversified such as Ger protein family to sense amino acids in *C. perfringens* and *C. botulinum* and CspC to sense taurocholate in *C. difficile*^34^. It is also known that *C. difficile* requires an amino acid signal, particularly glycine, as co-germinant of TCA, which may function through unknown receptor^36,37^. Glycine-conjugated BAs may interact with these cognate receptors as well as amino acid molecules and may efficiently trigger the germination of the wide range of gastro-intestinal tract bacteria . It warrants further study on the molecular mechanism of the signaling of glycine-conjugated BAs.

Previous large-scale isolation studies using TCA successfully isolated 105 novel species^7,8^. In this study, however, although on a smaller scale, most of the novel candidate species were isolated from the plate supplemented with glycine-conjugated BAs and only one was isolated from the TCA plate. This warrants a large-scale isolation study with varieties of BA molecules including novel conjugated BAs, such as phenylalanocholic acid, tyrosocholic acid and leucocholic acid, which were recently identified in human feces^38^.

In conclusion, our study showed the efficacy of a series of BAs for the cultivation and isolation of gastro-intestinal tract spore-forming bacteria including uncultured species. The efficacy depends on the BA signatures and the target bacterial species. A further study to address its molecular mechanism is warranted, in order to understand the BA-mediated gut microbial ecosystem as well as to capture unculturable microbiota.

## Materials and methods

### Sample collection

To test spore germination activity of BAs for gastro-intestinal tract bacteria, we collected fresh fecal samples from seven healthy adults aged from 22 to 32 years. Fresh feces were collected in two sterile tube (size 76 × 20 mm), each with 2 ml RNA later for 16S rRNA gene sequencing analysis or phosphate buffered saline (PBS) for bacterial culture. Collected samples in RNAlater were stored at 4 °C until further processing for DNA extraction. Collected samples in PBS were immediately processed within 1 h to preserve the viability of anaerobic bacteria.

### Bile acids

The BAs used in this study are: CA (Nacalai tesque, Japan), CDCA (Nacalai tesque), UDCA (Tokyo chemical industry Co. Ltd., Japan), DCA (Nacalai tesque), LCA (Nacalai tesque), their taurine conjugated forms, TCA (Tokyo Chemical Industry Co. Ltd.), TCDCA (FUJIFILM Wako Pure Chemical Co., Japan), taurodeoxycholic acid (TDCA, Nacalai tesque), taurolithocholic acid (TLCA, FUJIFILM Wako Pure Chemical Co.), tauroursodeoxycholic acid (TUDCA, Nacalai tesque), and their glycine conjugated forms, GCA (Tokyo Chemical Industry Co. Ltd.), GCDCA (Nacalai tesque), GDCA (Sigma-Aldrich, MO), glycolithocholic acid (GLCA, Nacalai tesque), glycoursodeoxycholic acid (GUDCA, Tokyo Chemical Industry Co. Ltd.). Stock solution of BAs was prepared by dissolving each BA in dimethyl sulfoxide to be 10% (w/v) except for TLCA, GLCA, TDCA, and GDCA to be 1% (w/v) followed by filter sterilization. Immediately before use, the BA solutions were diluted in reduced PBS to be 0.1% (w/v) except 0.01% (w/v) for the four BAs.

### Culturing

Bacteria culturing was performed under anoxic conditions in an anaerobic chamber (gas condition was 10% carbon dioxide, 10% hydrogen and 80% nitrogen). The fecal samples were homogenized in PBS (0.1 g stool per ml PBS), added by an equal volume of 70% (v/v) ethanol, and then incubated for 4 h at room temperature under ambient aerobic condition in order to kill vegetative cells^7^. Thereafter, the material was washed three times with PBS. Then, the ethanol-treated feces were resuspended with BA solutions (0.1 or 0.01 mg BAs per ml PBS) in order to stimulate spore germination and a spread on agar plate with Gifu-anaerobic medium (GAM, Nissui Pharmaceutical, Tokyo, Japan). For ethanol-untreated control, the fecal suspension was serial dilution and spread on the agar plates with GAM.

The inoculated agar plates were incubated for 72 h at 37 °C in the anaerobic chamber. The colonies formed on the agar medium were picked and inoculated in 3 ml GAM broth, and thereafter incubated overnight for the liquid culture. The cultured bacteria were reserved in glycerol stock at − 80 °C while analyzing the sequence of full-length 16S rRNA gene.

### 16S rRNA gene amplicon sequencing and analysis

All colonies on the plates were picked and suspended in 1 ml PBS by using inoculating loop. The genomic DNA was extracted from the pool by the beads-phenol method as previously described^39^. Bacterial DNA was also extracted from fecal samples using the same DNA extraction process. The extracted DNA was stored at − 30 °C until sequencing was performed on the Illumina MiSeq.

The variable region, V3-V4, of the 16S rRNA gene, was amplified from the extracted DNA as a template using the universal primers 341f. (5′- CGCTCTTCCGATCTCTGCCTACGGGNGGCWGCAG -3′) and 805r (5′- TGCTCTTCCGATCTGACGACTACHVGGGTATCTAATCC -3′), and TaKaRa Ex Taq HS (Takara Bio). The amplified products were then used as templates in a second PCR for further amplification using barcode-tagged primers. Both PCRs were performed as described previously^39^. PCR products were pooled and combined in equimolar amounts for sequencing using the Illumina MiSeq platform, generating 300 bp paired-end reads.

The sequences obtained from feces and cultured bacteria were processed using the Uparse pipeline in Usearch version 9.2 and 10.0. (https://drive5.com/useach/manual/upp_ill_pe.html).^40^Firstly, the pair of sequence reads were merged using the fastq_mergepairs script with mismatched windows up to 20 bases. After quality filtering, dereplication, the discard of singletons and length trimming, the merged sequences were clustered into 286 OTUs, each representing greater than 97% identity, using the UPARSE-OTU algorithm. The taxonomy of each OTU was assigned at phylum to species level using the EzBioCloud 16S rRNA gene database and the VSEARCH program^41^ and a cut-off of 0.97.

### Taxonomic classification of isolates

The taxonomy of the cultured isolates was analyzed using full-length 16S rRNA gene sequences. The full-length 16S rRNA gene fragment was amplified by PCR using the 27F (5′- AGAGTTTGATCCTGGCTCAG -3′) forward and 1497R (5′- GGTTACCTTGTTACGACTT -3′) reverse primers. Purified PCR products were subjected to Sanger sequencing by GENEWIZ (New jersey, USA). The resulting sequences were aligned using the Uparse/Usearch pipeline and were then grouped into OTUs, each representing greater than 98.7% sequence identity as per the UPARSE-OTU algorithm. The full-length 16S rRNA gene sequence of each species-level OTU was searched in the EzBioCloud 16S rRNA gene database (last updated Nov. 12th, 2019) to assign taxonomic designations to the species level and either known or novel candidate species status^31^. For phylogenetic analysis, all OTU sequences were aligned using ClustalW 2.1 (https://clustralw.ddbj.nig/) and then analyzed using the neighbor joining method^42^. For the neighbor-joining tree, evolutional distances were calculated based on a two-parameter model^43^. The rarefaction analysis was calculated by using the Usearch alpha_rare command.

### Statistical analysis

Statistical analyses were performed using the R package (V.3.2.2) (https://www.r-project.org/) and Excel 2011 (Microsoft). For comparing cfu among sample groups, the paired Wilcoxon singled-rank test was used. Cultured bacteria were clustered based on the relative abundance of OTUs cultured with each BA, in which hierarchical clustering was calculated based on complete linkage of the Eucidean distances matrix by the Heatmap.2 function in the R gplots package.

### Accession Number of 16S rRNA gene sequences

Raw sequence data were deposited in the DNA Data Bank of Japan (DDBJ) sequence read archive (DRA010323) under BioProject no. PRJDB012698, which contains links and access to stool sampling and cultured data under BioSample SAMD00230455 to SAMD00230551.

### Ethics approval

This study was approved by the Ethics Committees of the Faculty of Agriculture, Kyushu University (No. 19-006). All methods were carried out in accordance with the relevant guidelines and regulations. Written informed consent was obtained from donors of fecal samples. We entered and analyzed all samples anonymously and will publish all data anonymously using personal numbers.

## Supplementary information

Supplementary Information.
